# Supplementary material for: Reversal of high-glucose–induced transcriptional and epigenetic memories through NRF2 pathway activation
Source: Life Sci Alliance. 2024 May 16;7(8):e202302382. doi: 10.26508/lsa.202302382 (PMC11099870; doi:10.26508/lsa.202302382)
Supplement: Supplementary file 3 [file LSA-2023-02382_TableS1.docx]

**Supplemental Table 1.** Summary of sequencing and mapping of RNA-seq samples.

| **Sample name** | **Sequenced pairs of reads (millions)** | **Mapped reads (millions)** |
| --- | --- | --- |
| Control A | 21.5 | 19 |
| Control B | 21.3 | 19.9 |
| Control C | 62.4 | 58.4 |
| High glucose A | 69.8 | 65.4 |
| High glucose B | 44.4 | 41.7 |
| High glucose C | 50.2 | 46.5 |
| Memory A | 57.4 | 52.9 |
| Memory B | 32.3 | 30 |
| Memory C | 48.5 | 44.9 |
| NRF2-OE HG_A | 26.3 | 24.6 |
| NRF2-OE HG_B | 16.7 | 15.6 |
| NRF2-OE HG_C | 14.3 | 13.3 |
| NRF2-OE Memory A | 39.2 | 36.8 |
| NRF2-OE Memory B | 20.4 | 18.6 |
| NRF2-OE Memory C | 27.9 | 25.6 |
| SF HG_A | 69 | 64.6 |
| SF HG_B | 20 | 18.4 |
| SF HG_C | 34.7 | 32.4 |
| SF memory A | 24.8 | 22.6 |
| SF memory B | 31.1 | 29 |
| SF memory C | 22.4 | 20.5 |
