# Supplementary material for: Reversal of high-glucose–induced transcriptional and epigenetic memories through NRF2 pathway activation
Source: Life Sci Alliance. 2024 May 16;7(8):e202302382. doi: 10.26508/lsa.202302382 (PMC11099870; doi:10.26508/lsa.202302382)
Supplement: Supplementary file 4 [file LSA-2023-02382_TableS2.docx]

**Supplemental Table 2.** Summary of sequencing and mapping of ATAC-seq samples.

| **Sample name** | **Sequenced pairs of reads (millions)** | **Mapped fragments (millions)** |
| --- | --- | --- |
| Control A | 39.5 | 37 |
| Control B | 51.5 | 49.3 |
| High glucose A | 40.1 | 38 |
| High glucose B | 38.4 | 36.6 |
| Memory A | 43.5 | 41.3 |
| Memory B | 37.2 | 35.6 |
| Memory + SF A | 31.6 | 30.9 |
| Memory + SF B | 32.5 | 31.7 |
