# Supplementary material for: Reversal of high-glucose–induced transcriptional and epigenetic memories through NRF2 pathway activation
Source: Life Sci Alliance. 2024 May 16;7(8):e202302382. doi: 10.26508/lsa.202302382 (PMC11099870; doi:10.26508/lsa.202302382)
Supplement: Supplementary file 5 [file LSA-2023-02382_TableS3.docx]

**Supplemental Table 3.** List of primers used for RT-qPCR

| **Name (F is forward primer and R is reverse primer)** | **Sequence (5’-3’)** |
| --- | --- |
| TGFB2_F | CAGCACACTCGATATGGACCA |
| TGFB2_R | CCTCGGGCTCAGGATAGTCT |
| Actin_F | GCTATCCAGGCTGTGCTATC |
| Actin_R | TGAGGTAGTCAGTCAGGTCC |
| NQO1_F | GAAGAGCACTGATCGTACTGGC |
| NQO1_R | GGATACTGAAAGTTCGCAGGG |
| HMOX1_F | GACCCATGACACCAAGGACC |
| HMOX1_R | TCCACGGGGGCAGAATCTTG |
| PPARG_F | ACCAAAGTGCAATCAAAGTGGA |
| PPARG_R | ATGAGGGAGTTGGAAGGCTCT |
| RB1_F | TTGGATCACAGCGATACAAACTT |
| RB1_R | AGCGCACGCCAATAAAGACAT |
| ANPEP_F | TTCAACATCACGCTTATCCACC |
| ANPEP_R | AGTCGAACTCACTGACAATGAAG |
| FST_qF | TCTGCCAGTTCATGGAGGA |
| FST_qR | TCCTTGCTCAGTTCGGTCTT |
| NFE2L2_F | TCCAGTCAGAAACCAGTGGAT |
| NFE2L2_R | GAATGTCTGCGCCAAAAGCTG |
| TNFSF10 F | TGCGTGCTGATCGTGATCTTC |
| TNFSF10 R | GCTCGTTGGTAAAGTACACGTA |
